# Glucocorticoid receptor antagonism promotes apoptosis in solid tumor cells

**DOI:** 10.18632/oncotarget.27989

**Published:** 2021-06-22

**Authors:** Andrew E. Greenstein, Hazel J. Hunt

**Affiliations:** ^1^Corcept Therapeutics, Menlo Park, CA, USA

**Keywords:** glucocorticoid, tumors, apoptosis, chemotherapy, drug resistance

## Abstract

Background: Resistance to antiproliferative chemotherapies remains a significant challenge in the care of patients with solid tumors. Glucocorticoids, including endogenous cortisol, have been shown to induce pro-survival pathways in epithelial tumor cells. While pro-apoptotic effects of glucocorticoid receptor (GR) antagonism have been demonstrated under select conditions, the breadth and nature of these effects have not been fully established.

Materials and Methods: To guide studies in cancer patients, relacorilant, an investigational selective GR modulator (SGRM) that antagonizes cortisol activity, was assessed in various tumor types, with multiple cytotoxic combination partners, and in the presence of physiological cortisol concentrations.

Results: In the MIA PaCa-2 cell line, paclitaxel-driven apoptosis was blunted by cortisol and restored by relacorilant. In the OVCAR5 cell line, relacorilant improved the efficacy of paclitaxel and the potency of platinum agents. A screen to identify optimal combination partners for relacorilant showed that microtubule-targeted agents consistently benefited from combination with relacorilant. These findings were confirmed in xenograft models, including MIA PaCa-2, HeLa, and a cholangiocarcinoma patient-derived xenograft. *In vivo*, tumor-cell apoptosis was increased when relacorilant was added to paclitaxel in multiple models.

Conclusions: These observations support recently reported findings of clinical benefit when relacorilant is added to paclitaxel-containing therapy in patients with ovarian and pancreatic cancers and provide a new rationale for combining relacorilant with additional cytotoxic agents.

## INTRODUCTION

Drug resistance, whether primary or acquired, is a major impediment to cancer therapy. For both targeted agents and older chemotherapies, initial reductions in tumor volume typically have limited duration and ultimately are followed by the growth of drug-tolerant tumors. Multiple mechanisms of drug resistance have been proposed [1–3], including outgrowth of pre-existing clones with a drug-resistance mutation, epithelial to mesenchymal transition, or epigenetic reprogramming leading to drug-tolerant persister (DTP) cells. Glucocorticoids (GC), including endogenous cortisol, have also been shown to induce pro-survival pathways in epithelial tumor cells [4–6].

Cortisol is a ubiquitous and essential hormone that agonizes the glucocorticoid receptor (GR). Elevated cortisol activity has been implicated in the pathophysiology of multiple cancer types, but even normal physiological levels achieve GR agonism [7]. Normal serum cortisol concentrations range from 276–552 nM. GR agonism could contribute to tumor cell biology even in patients with normal cortisol levels.

The GR is expressed in nearly every cell of the body [8, 9]. Through variation in upstream co-regulators and downstream epigenetics, GR activation has dramatically different consequences on the physiology of distinct cell types [7]. GR agonists, including cortisol, have demonstrated pro-apoptotic effects in hematological malignancies, cytostatic effects on sarcoma-derived cell lines, and anti-apoptotic effects in carcinoma cell lines [10–13]. GR antagonism has demonstrated pro-apoptotic effects in ovarian and breast cancer cell lines [14–16]. *In vitro* studies suggest suppression of *sgk1* and *dusp1* expression, both of which control cell survival pathways, may be involved in GR antagonist pro-apoptotic effects [15]. To guide the clinical application of this mechanism, additional studies were necessary to explore other epithelial tumor types, optimal chemotherapy combination partners, and GR antagonist effects under conditions mimicking endogenous GC concentrations. Here, we present the findings of a panel of these studies using the investigational, potent selective GR modulator (SGRM) relacorilant (Corcept Therapeutics).

GR binding of relacorilant was observed with a K_i_ of 0.15 nM, while no measurable binding was detected with the progesterone, estrogen, or androgen receptors [17]. Unlike the non-specific steroidal GR antagonist mifepristone (Korlym®, Corcept Therapeutics), relacorilant does not exhibit partial agonist activity toward human or mouse GR. Mifepristone and relacorilant are both competitive antagonists of the GR and are best studied in the context of physiologically relevant cortisol concentrations. Relacorilant, administered orally at doses that achieved systemic exposure similar to those seen in phase 1 studies, normalized glucose and insulin in a rat model of cortisone-induced insulin resistance [17]. Phase 1 studies in healthy volunteers demonstrated tolerability and the ability to blunt the pharmacodynamic effects of a single dose of prednisone [18, 19] (NCT03508635). In a phase 2 study in patients with Cushing syndrome, relacorilant demonstrated the ability to reverse the effects of excess cortisol on hypertension and insulin resistance [20], and it is currently being studied in two phase 3 trials in patients with endogenous Cushing syndrome, GRACE (NCT03697109) and GRADIENT (NCT04308590).

In the studies presented here, we assessed the effects of relacorilant on tumor cells in the presence of anti-proliferative and cytotoxic agents. The effect of relacorilant on viability and apoptosis in multiple solid tumor types was explored *in vitro* and *in vivo*.

## RESULTS

### Cortisol suppresses and relacorilant restores apoptotic effects of paclitaxel *in vitro*

To interpret the effects of relacorilant in combination with cytotoxic therapy, the effects of GC alone first needed to be characterized. Solid-tumor cell lines whose growth was affected by GC were identified (Table 1). GC increased the viability of three cell lines, suggesting that a GR antagonist might decrease the viability of those lines independent of cytotoxic agent activity. Such cell lines were excluded from further study as the potentiation of the cytotoxic agent could not be easily distinguished from direct effects on viability. Cell lines like OVCAR5, in contrast, provide a more interpretable model to investigate the effects of GC on cytotoxic agent activity because GC alone did not significantly affect growth. To avoid interference from GCs in FBS, FBS was diluted to 2.5% v/v or charcoal-dextran stripped FBS (CDS-FBS, in which the GCs are removed) was used. Similar effects of GC were observed across various tissues of origin and in both standard 2.5% FBS and CDS-FBS. These initial experiments were conducted using a viability assay (CellTiter-Glo), and thus effects on growth rate could not be distinguished from effects on apoptosis.

**Table 1:** Effects of GC (100 nM dexamethasone or 400 nM cortisol) on cell line growth

Cell line	Type	Media	Growth, as a % of no-GC control	Parametric *T-*test
H2122	Lung	FBS	47	**0.00001**
BxPC3	Pancreatic	FBS	74	**0.017**
RT-112	Bladder	FBS	75	**0.0002**
BxPC3	Pancreatic	CDS	75	**0.019**
AsPC1	Pancreatic	CDS	79	**0.001**
MIA PaCa-2	Pancreatic	CDS	79	0.376
SU8686	Pancreatic	CDS	83	0.100
AsPC1	Pancreatic	FBS	83	**0.0001**
MIA PaCa-2	Pancreatic	FBS	86	**0.002**
SU8686	Pancreatic	CDS	86	**0.033**
RT112	Bladder	FBS	86	**0.0003**
IMR32	Neuroblastoma	FBS	92	0.353
H460	Lung	FBS	95	0.104
OVCAR3	Ovarian	CDS	99	0.817
ZR-75	HR+BC	FBS	99	0.565
HT144	Melanoma	FBS	100	0.847
H1703	Lung	FBS	101	0.893
OVCAR3	Ovarian	FBS	101	0.560
SU8686	Pancreatic	FBS	103	0.399
G361	Melanoma	FBS	103	0.407
HeLa	Cervical	FBS	103	0.166
MCF7	HR+BC	FBS	104	0.409
SU8686	Pancreatic	FBS	104	0.181
G361	Melanoma	FBS	105	0.244
OVCAR5	Ovarian	FBS	105	0.364
OVCAR5	Ovarian	CDS	106	0.169
CAPAN1	Pancreatic	FBS	107	**0.001**
CAPAN1	Pancreatic	CDS	108	**0.005**
HSC-3	OSCC	FBS	111	**0.034**
H1975	Lung	FBS	117	**0.003**

Significant differences in cell growth with or without GC (unpaired parametric *T*-test *P* < 0.05) highlighted in Bold. Growth, as a % of no-GC control = (Viability in the presence of 400 nM cortisol or 100 nM dexamethasone)/(Viability in media lacking GC) × 100. Abbreviations: BC, breast cancer; CDS, charcoal-dextran stripped; FBS, fetal bovine serum; GC, glucocorticoid; HR+, hormone receptor positive; OSCC, oral squamous cell carcinoma.

**Figure 1:**
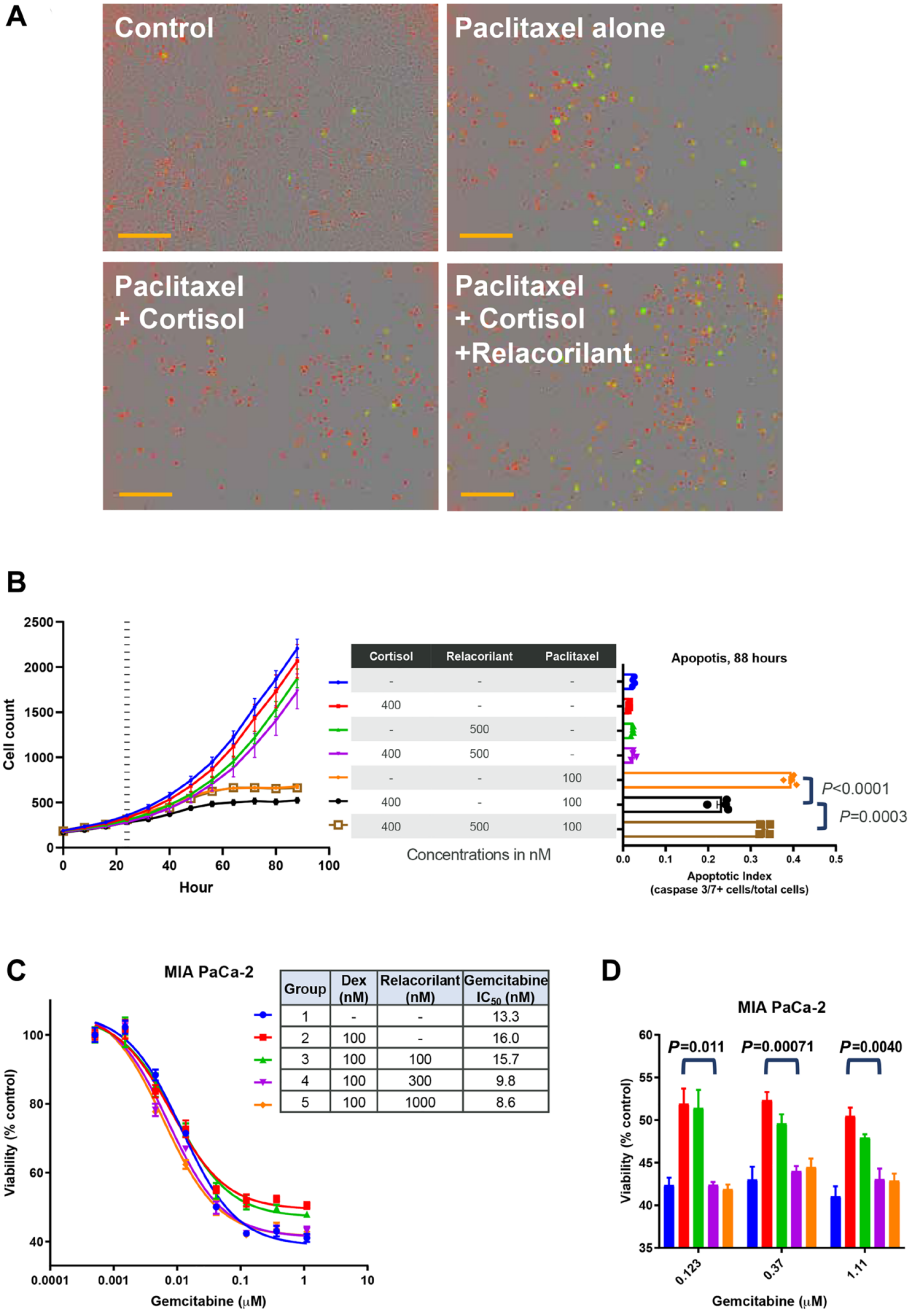
Apoptotic effects of paclitaxel and gemcitabine are suppressed by cortisol and restored by relacorilant *in vitro*. (**A**) MIA PaCa-2 cells were grown in the presence of 100 nM paclitaxel, 100 nM paclitaxel + 400 nM cortisol, or 100 nM paclitaxel + 400 nM cortisol + 500 nM relacorilant and compared to an untreated control. Nuclear dye (red) was used to quantify cells, and apoptosis was assessed using a fluorescent caspase 3/7 substrate peptide. Scale bars (orange) represent 300 μm. (**B**) Images of cells (NucLight Red, left) and an apoptosis reporter (Caspase 3/7 substrate, right) were quantified under the treatment conditions (100 nM paclitaxel, 400 nM cortisol, 500 nM relacorilant). Cortisol/relacorilant were added at time 0 and paclitaxel was added 24 hours later (vertical dashed line on the left). Peak apoptosis occurred at 88 hours, corresponding to 64 hours after paclitaxel addition. Cortisol reduced apoptosis by paclitaxel (unpaired parametric *T*-test *P* < 0.0001) and relacorilant increased apoptosis (unpaired parametric *T*-test *P* = 0.0003). (**C**) Gemcitabine was titrated in the presence of glucocorticoid receptor agonist (dexamethasone, 100 nM) or antagonist (relacorilant, 100, 300, or 1000 nM) and cell viability was assessed 72 hours later (CellTiter-Glo). A modest, dose-dependent improvement in gemcitabine potency was observed. (**D**) At high doses of gemcitabine (>0.1 μM), dexamethasone consistently reduced the efficacy of gemcitabine. This effect was reversed by doses of relacorilant in excess of the dexamethasone concentration. Unpaired non-parametric *T*-test *p-value*s shown comparing gemcitabine+dexamethasone to gemcitabine+dexamethasone+relacorilant (300 nM) groups. Abbreviations: CORT, cortisol; DEX, dexamethasone; GEM, gemcitabine; GR, glucocorticoid receptor; h, hours; NS, not significant; PAC, paclitaxel; RELA, relacorilant.

To assess the effects of relacorilant + cytotoxic therapy on proliferation rate and apoptosis, the MIA PaCa-2 cell line was assessed in a kinetic apoptosis assay. Cell counts were quantified using a nuclear dye, and apoptosis was monitored using a fluorescent caspase 3/7 substrate (Figure 1A). Cortisol 400 nM (a concentration within the normal human serum range [21]) alone or in combination with 100 nM paclitaxel resulted in a non-significant trend toward reduced proliferation rate (Figure 1B, left). Paclitaxel alone induced apoptosis, while the addition of 400 nM cortisol significantly reduced apoptosis (Figure 1B, right). Relacorilant reversed the effects of cortisol and partially restored paclitaxel’s induction of tumor-cell apoptosis.

To explore the breadth of these pro-apoptotic effects of relacorilant in combination with paclitaxel, the MIA PaCa-2 cells were next assessed with distinct cytotoxic agents and dexamethasone, a GR agonist. Using 100 nM dexamethasone, MIA PaCa-2 viability was assessed using a viability (CellTiter-Glo) endpoint assay across a range of gemcitabine combinations. Dexamethasone numerically increased the IC_50_ (i.e., reduced potency) of gemcitabine (Figure 1C), though the effect was non-significant. This modest effect was dose-dependently reversed by adding relacorilant (Figure 1C), while the effect was again non-significant. Dexamethasone increased the residual viability (i.e., reduced the maximal efficacy) of high doses (> 0.1 μM) of gemcitabine (Figure 1D). Relacorilant alone had no off-target, agonist-independent activity, which is consistent with the mechanism of a competitive antagonist neutralizing the activity of an agonist (dexamethasone or cortisol). Both cortisol, used to model endogenous GR agonism, and dexamethasone, used to assess a more GR-specific agonist were assessed in these experiments. The data in Figure 1 demonstrates that both agonists can promote MIA PaCa-2 cell survival in the presence of distinct cytotoxic agents and relacorilant can reverse these effects.

**Figure 2:**
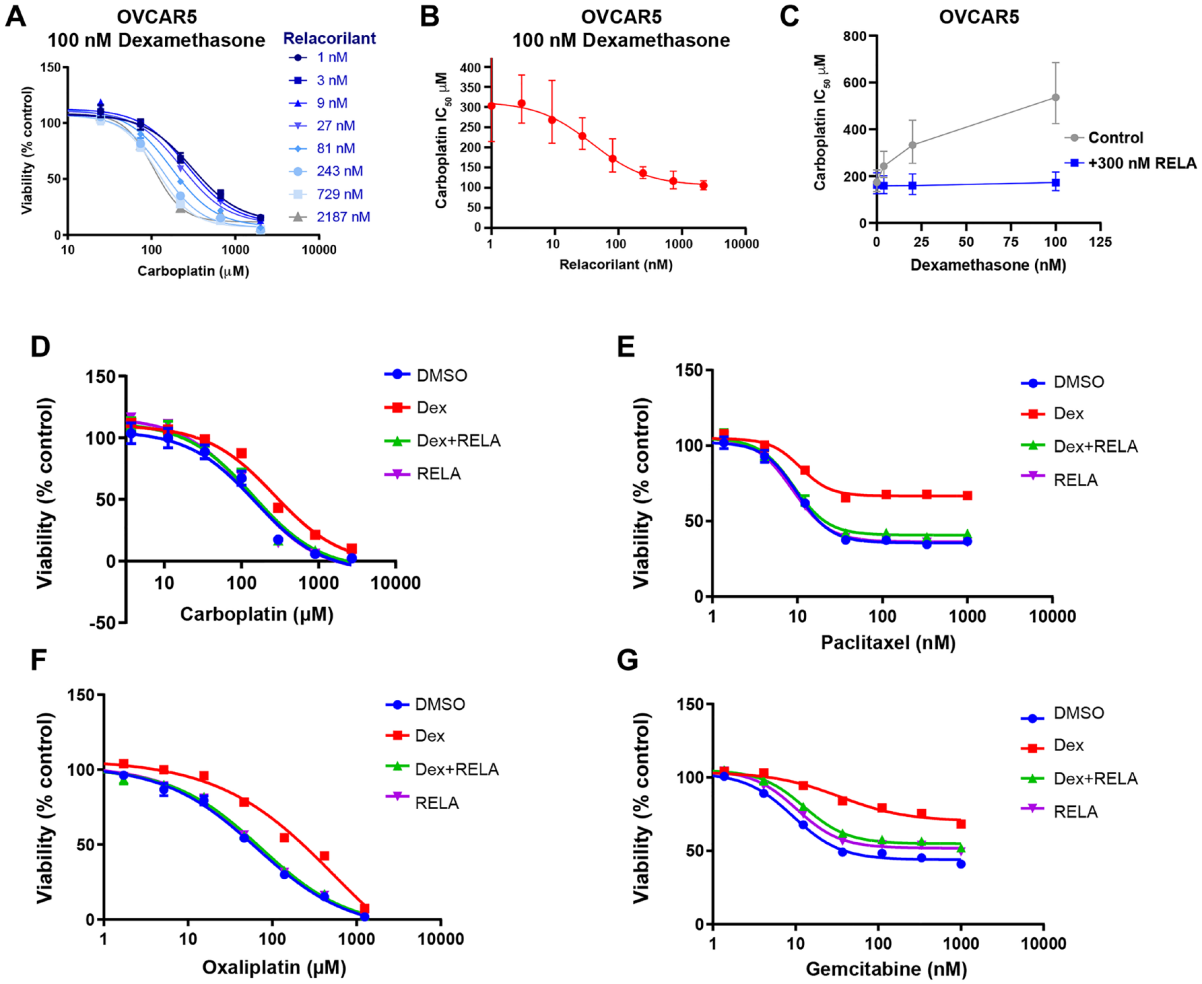
Potency and efficacy of cytotoxic agents are improved by relacorilant in OVCAR5 cells. (**A**) Carboplatin was titrated in the presence of 100 nM dexamethasone and increasing concentrations of relacorilant. Viability was assessed using CellTiter-Glo. Errors bars represent standard error across quadruplicate measurements. (**B**) Increasing potency of carboplatin (decreasing IC_50_) was observed as a function of relacorilant concentrations in the presence of 100 nM dexamethasone. Error bars represent 95% confidence limits of IC_50_ values. (**C**) Conversely, increasing dexamethasone diminished the potency of carboplatin. This effect is ablated in the presence of relacorilant. Error bars represent 95% confidence limits of IC_50_ values. (**D**–**G**) Dose-response curves for carboplatin, oxaliplatin, paclitaxel, and gemcitabine are shown under conditions including 100 nM dexamethasone and/or 300 nM relacorilant. Error bars show the standard error. Abbreviations: DEX, dexamethasone; DMSO, dimethyl sulfoxide; RELA, relacorilant.

To confirm the dose dependency of the observed GR agonist and antagonist effects, dexamethasone or relacorilant were titrated while cell viability was assessed. OVCAR5 cells were used to extend findings into cells of distinct tissue of origin, and carboplatin was chosen given its use in ovarian cancer clinical practice. In the presence of 100 nM dexamethasone, carboplatin potency was improved by increasing concentrations of relacorilant (Figure 2A). The IC_50_ of carboplatin was calculated at each concentration of relacorilant and plotted to demonstrate the dose-dependent effect of relacorilant on carboplatin potency (Figure 2B). Conversely, the carboplatin potency was diminished by increasing concentrations of dexamethasone (Figure 2C). The effect of dexamethasone on carboplatin potency was eliminated by the presence of relacorilant (Figure 2C). These data support on-target, GR-mediated, dose-dependent effects of relacorilant on carboplatin potency.

To further explore the differential effects of GR agonists and antagonists on the potency or maximal efficacy of a cytotoxic agent, titrations of additional cytotoxic agents in the presence of 100 nM dexamethasone and 300 nM relacorilant were assessed. Dexamethasone diminished and relacorilant restored the potency (IC_50_) of carboplatin and oxaliplatin (Figure 2D–2G and Table 2). Dexamethasone and relacorilant had a small but significant effect on carboplatin and oxaliplatin efficacy, quantified as the residual percent of viable cells at the maximum dose after 72 hours (Figure 2D–2G and Table 2). In contrast, dexamethasone and relacorilant had a larger and significant effect on paclitaxel efficacy without a clear effect on potency (Figure 2D–2G and Table 2). Dexamethasone and relacorilant affected both potency and efficacy of gemcitabine (Figure 2D–2G and Table 2). Together, these observations suggest that GR effects on potency may be distinct from the efficacy effects depending on the type of the cytotoxic agent.

**Table 2:** Potency and efficacy of cytotoxic agents in OVCAR5 cells

Cytotoxic agent	Metric	Control	DEX	DEX + RELA	RELA
**Carboplatin**	IC_50_ (μM)	150	266	141	129
	IC_50_ 95% confidence limits (μM)	99.2 to 225.6	203.1 to 349.2	104.5 to 190.9	103.7 to 160
	Residual viability (%)	2	10	5	3
	Residual viability unadjusted *p*-value vs DEX group	0.0019	NA	< 0.01	< 0.001
**Oxaliplatin**	IC_50_ (μM)	65	566	72	71
	IC_50_ 95% confidence limits (μM)	48.2 to 97.8	238.2 to 5767	56.2 to 101.9	59.8 to 88.8
	Residual viability (%)	2	7	3	2
	Residual viability unadjusted *p*-value vs DEX group	0.0116	NA	0.0165	0.0109
**Paclitaxel**	IC_50_ (nM)	10	11	9	9
	IC_50_ 95% confidence limits (nM)	8.5 to 11.4	8.5 to 13.2	7.6 to 10.9	8 to 9.6
	Residual viability (%)	36	67	41	36
	Residual viability unadjusted *p*-value vs DEX group	< 0.001	NA	< 0.001	< 0.001
**Gemcitabine**	IC_50_ (nM)	9	34	13	10
	IC_50_ 95% confidence limits (nM)	8.1 to 10.7	21 to 71.9	10.3 to 15.6	9.2 to 11.5
	Residual viability (%)	44	70	55	52
	Residual viability unadjusted *p*-value vs DEX group	< 0.001	NA	< 0.001	< 0.001

Residual viability are at top concentration of cytotoxic agent tested: 2700 μM carboplatin, 1250 oxaliplatin μM, and 1000 nM for both paclitaxel and gemcitabine. Unpaired non-parametric *T*-tests compared the residual viability at the maximum cytotoxic agent concentration between each group and the DEX-treated group. Abbreviations: DEX, dexamethasone; NA, not applicable; RELA, relacorilant.

### Relacorilant improves efficacy of microtubule inhibitors

**Figure 3:**
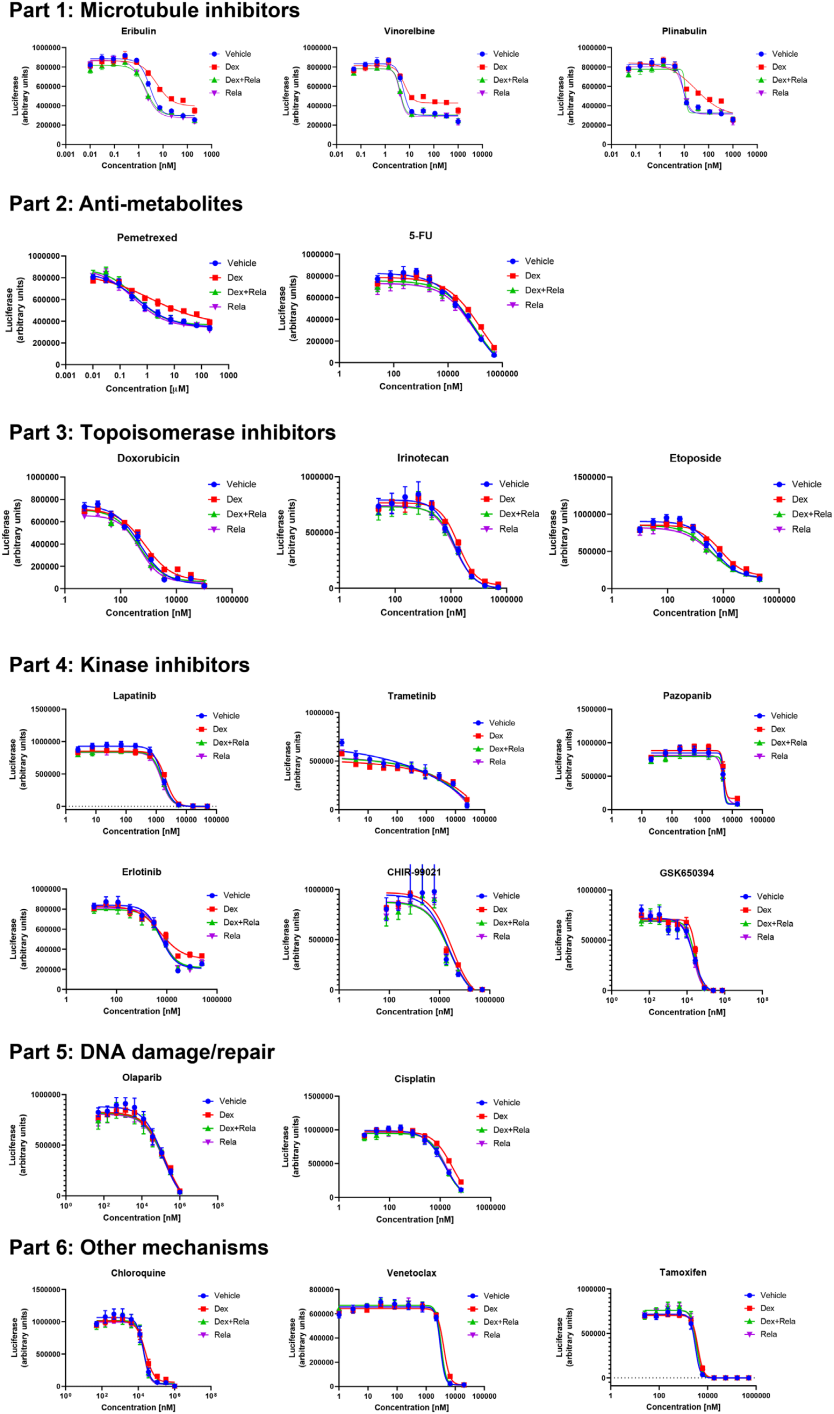
Effects of relacorilant on cytotoxic agent activity in OVCAR5 cells. Dose-response curves for distinct classes of cytotoxic agents. Conditions included control, 100 nM dexamethasone, 300 nM relacorilant, or 100 nM dexamethasone + 300 nM RELA. Error bars show the standard error. Abbreviations: DEX, dexamethasone; RELA, relacorilant.

The observations in Figure 2 and Table 1 raised the possibility that the effects of relacorilant on a cytotoxic agent’s potency or efficacy could be related to the cytotoxic agent’s mechanisms of action. Viability of OVCAR5 cells was initially tested with 19 cytotoxic agents alone to determine baseline potency and efficacy (data not shown). Subsequently, the dose response for each agent was conducted with GR agonist, GR antagonist, both, or neither. Relacorilant improved IC_50_, residual viability, or both for multiple agents (Figure 3). Microtubule inhibitors showed a consistent benefit from combination with relacorilant in the OVCAR5 cell line.

### Relacorilant increases apoptotic activity of cytotoxic therapy in xenograft models

**Figure 4:**
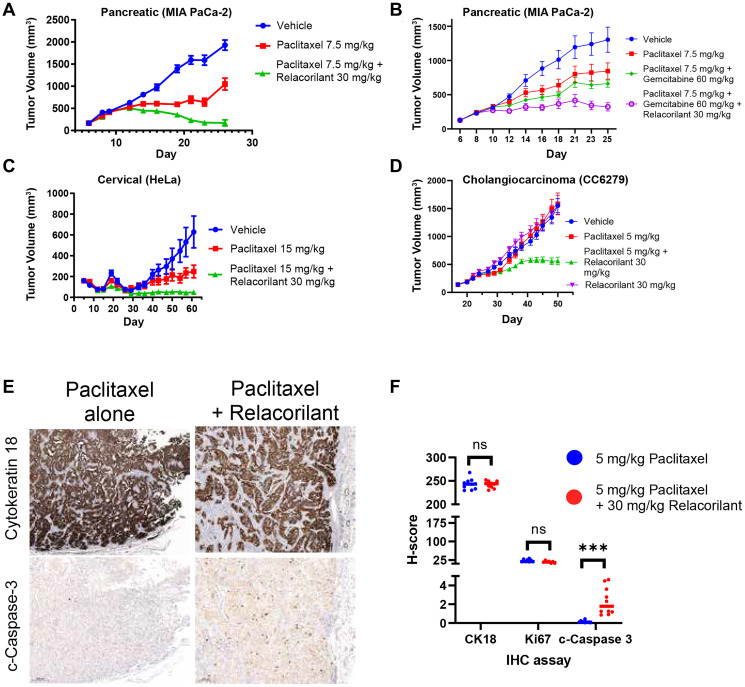
Relacorilant improves the efficacy and promotes apoptotic activity of cytotoxic therapy in xenograft models under physiological cortisol conditions. (**A**) In the MIA PaCa-2 model, efficacy of the combination of paclitaxel + relacorilant was significantly better than paclitaxel alone (non-parametric *T*-test *P* < 0.0001). (**B**) In the MIA PaCa-2 model, efficacy of paclitaxel + gemcitabine + relacorilant was better than paclitaxel + gemcitabine alone (non-parametric *T*-test *P* = 0.0005). (**C**) In the HeLa model, efficacy of paclitaxel + relacorilant was significantly better than paclitaxel alone (non-parametric *T*-test *P* < 0.0001). (**D**) In the CC6279 model, efficacy of paclitaxel + relacorilant was significantly better than paclitaxel alone (non-parametric *T*-test *P* < 0.0001). Relacorilant alone had no significant effect on tumor growth. Error bars represent the standard error; all studies 10 animals/group. (**E**) Tumor cells were labeled using cytokeratin 18 immunohistochemistry (top). In serial sections, apoptotic caspase activity (cleaved caspase 3, bottom) and proliferation (Ki67, not shown) were assessed. (**F**) Relacorilant increased the cleaved caspase intensity and prevalence (H-score) within the tumor cells compared to paclitaxel alone. ^***^Mann-Whitney, *P* < 0.0001. Abbreviation: IHC, immunohistochemistry.

To assess the effects of relacorilant under physiological GC conditions, xenograft studies were conducted. The mice were treated and handled to avoid altering normal GC levels, which in the mouse are primarily corticosterone levels, by allowing extra time for acclimatization and minimizing handling. Consistent with the *in vitro* results, relacorilant improved the efficacy of paclitaxel in the MIA PaCa-2 xenograft (Figure 4A). Because gemcitabine + paclitaxel is an approved standard of care in pancreatic cancer, relacorilant benefit on top of gemcitabine + paclitaxel was also assessed (Figure 4B). Gemcitabine + paclitaxel was more effective than paclitaxel alone, and relacorilant further improved the efficacy of gemcitabine + paclitaxel. To extend these findings, xenografts from distinct tissues of origin were also assessed. Both the HeLa (cervical) xenograft and CC6279 (a patient-derived cholangiocarcinoma xenograft) showed significant improvements in the efficacy of paclitaxel when relacorilant was added (Figure 4C and 4D). As expected from the *in vitro* results, relacorilant alone had no effects in the CC6279 models (Figure 4D) and was thus not included in other xenograft studies as it would be unethical and unnecessary to repeatedly confirm this. Together, these data demonstrate that relacorilant can improve the efficacy of chemotherapies *in vivo*.

The initial *in vitro* observations suggested that an increase in apoptosis, rather than a decrease in proliferation rate, was achieved when relacorilant was added to a cytotoxic therapy (Figure 1). To determine if this was recapitulated *in vivo*, apoptosis markers were assessed in relacorilant-treated xenografts. The CC6279 cholangiocarcinoma model was assessed because the relacorilant effect size was greatest. Cleaved caspase 3 activity was qualitatively and quantitatively elevated in mice treated with relacorilant + paclitaxel compared to paclitaxel alone, while no difference was observed for CK18 (a tumor cell marker) and Ki67 (a proliferation marker) (Figure 4E and 4F). Consistent with the initial *in vitro* observation, relacorilant promoted tumor cell apoptosis in xenograft models.

## DISCUSSION

The findings reported here provide new insights into mechanisms of resistance to anti-proliferative agents. First, when combined with a cytotoxic agent, GR antagonism can result in improved potency, efficacy, or both of the cytotoxic agent. Second, GR antagonism improves the potency or efficacy of cytotoxic agents with diverse mechanisms and is particularly promising when combined with microtubule-targeting cytotoxic agents. Third, GR antagonism enhances paclitaxel-induced apoptosis in multiple types of tumor cells, including pancreatic and cholangiocarcinoma-derived cells. Fourth, GR agonists and antagonists have opposite and dose-dependent effects on tumor cell viability. Finally, GR antagonism enhances tumor cell apoptosis due to cytotoxic agents under physiological GC concentrations in mice. These new findings will help guide the clinical development of relacorilant in combination with cytotoxic agents for the treatment of solid tumors.

Anti-proliferative agents, including, but not limited to, paclitaxel, consistently cause tumor cell apoptosis *in vitro*. Resistance to such therapies is, unfortunately, the norm in clinical practice in patients with solid tumors. The data presented here expand our understanding of the pro-apoptotic effects of GR antagonists described in ovarian and breast cancer cells [14–16]. Anti-apoptotic effects of GR agonism were demonstrated in pancreatic, bone, brain, breast, cervix, melanoma, and neuroblastoma cancer cells when combined with a fixed concentration of paclitaxel, cisplatin, or 5-FU [5, 22]. Building on this, pro-apoptotic effects of GR antagonism were demonstrated with cisplatin and gemcitabine in pancreatic cancer cells [6], paclitaxel in breast cancer cells [14, 23], carboplatin in ovarian cancer cells [15], and docetaxel in prostate cancer cells [24]. Most of these studies [6, 14, 24] used the promiscuous hormone receptor antagonist RU-486 (mifepristone). The work described in this manuscript, including a more specific GR antagonist and dose-dependent comparisons of multiple classes of cytotoxic therapy, was intended to guide translation of these foundational observations into a clinically actionable context for a phase 1 study.

Relacorilant improved the activity of diverse cytotoxic agents, and the most pronounced benefits were seen in combination with microtubule-targeted agents. Other reports suggest possible mechanisms by which GR-mediated transcription could interact with apoptotic signaling, particularly for this class of agents [24]. Mechanism of microtubule-inhibitor-mediated apoptosis requires phosphorylation of BCL2 by JNK, which diminishes the anti-apoptotic activity of BCL2 [25]. GR agonism increases DUSP1 transcription [14], DUSP1 dephosphorylates and inactivates JNK, and BLC2 phosphorylation is reduced, which maintains its anti-apoptotic activity. Additionally, modulation of the expression of RANBP1, a key regulator of mitotic spindle function, by SGK1, a GR-controlled gene [14], has been suggested as a mechanism linking GR agonism to resistance toward microtubule-targeted agents [26]. Both mechanisms, or additional yet-undescribed pathways, may be responsible for the observed benefits of combining a microtubule inhibitor with a GR antagonist. Further, relacorilant improved the efficacy and/or potency of other commonly prescribed cytotoxic therapies, including, but not limited to, carboplatin and gemcitabine, which provides a rationale for the clinical assessment of these agents in combinations with relacorilant.

Targeting the origins of drug-resistant cancer cells via GR antagonism is an early but promising approach to lengthening clinical benefit of cytotoxic therapies. DTP cells are a sub-population of tumor cells that survive a dose of cytotoxic agent which is lethal to most of that population [3]. The ‘efficacy’ and ‘residual viability’ effects reported in Tables 1 and 2 are a direct measure of DTP abundance. GR activity may underly the switch into the drug tolerant state by modifying the biological processes implicated in DTP emergence. GR activity has been demonstrated to affect such processes, including transcriptional reprograming, epigenetics, epithelial to mesenchymal transition, and TGFβ activity [23, 27–29]. Proposed DTP targets KDM5A and GPX4 [3, 30] are like GR in their heterogeneous and reversible involvement in these processes. Heterogeneity in GR co-regulator expression within a population of tumor cells could prime a sub-population to survive an otherwise apoptotic dose of paclitaxel. Given the safety and tolerability established with relacorilant in phase 1–3 trials, clinical translation of this mechanism could be more expedient than other proposed targets. While physiological cortisol is ubiquitous (normal range is 276–552 nM in human serum), standard tissue culture conditions employ low or no cortisol (carried over only at low levels in bovine serum) and thus cortisol involvement in DTPs had not been appreciated previously.

Clinical application of this information also requires key considerations involving the use of exogenous GR agonists (e.g., prednisone and dexamethasone) in clinical cancer care [31]. These agents are routinely used to mitigate hypersensitivity reactions to cremophor (a component of paclitaxel formulation), suppress nausea, reduce swelling in cerebral malignances, and reverse immune related adverse events that commonly arise with CTLA-4 and PD-(L)1 targeted therapies. Thus, the optimal clinical application for a GR antagonist would necessarily avoid the use of corticosteroids. Nab-paclitaxel (Abraxane^®^), paclitaxel formulated with albumin and without cremophor, does not require co-administration of corticosteroids and is thus a rational first choice for combination with relacorilant, given the data presented here. This rationale supports two trials of relacorilant in combination with nab-paclitaxel, a phase 2 trial in ovarian cancer (NCT03776812) and a phase 3 trial in pancreatic cancer (NCT04329949). Additionally, the benefit of relacorilant combined with a diverse set of cytotoxic agents identified here further expands the number of potential corticosteroid-sparing regimens with relacorilant.

## MATERIALS AND METHODS

### Cell line authentication and *in vitro* cell culture

Cell lines were supplied by Charles River Labs directly or via ATCC (NCI-H2122 [RRID:CVCL_1531], BxPC-3 [RRID:CVCL_0186], RT-112 [RRID:CVCL_1670], AsPC-1 [RRID:CVCL_0152], MIA PaCa-2 [RRID:CVCL_0428], SU.86.86 [RRID:CVCL_3881], RT-112 [RRID:CVCL_1670], IMR-32 [RRID:CVCL_0346], NCI-H460 [RRID:CVCL_0459], OVCAR-3 [RRID:CVCL_0465], ZR-75-1 [RRID:CVCL_0588], HT-144 [RRID:CVCL_0318], NCI-H1703 [RRID:CVCL_1490], G-361 [RRID:CVCL_1220], HeLa [RRID:CVCL_0030], MCF-7 [RRID:CVCL_0031], OVCAR-5 [RRID:CVCL_1628], Capan-1 [RRID:CVCL_0237], HSC-3 [RRID:CVCL_1288], NCI-H1975 [RRID:CVCL_1511]). Genetica Cell Line Testing (LabCorp) was used for cell line authentication, with PowerPlex^®^ 16 HS (Promega Corporation) Multiplex STR technology providing high-resolution typing with 15 autosomal loci and 1 gender determination marker. Tests included a mouse marker for the detection of mouse DNA. Expression of GR was confirmed using public data from the Cancer Cell Line Encyclopedia [32]. Cells were grown in the recommended base media and supplemented with 2.5% fetal bovine serum (FBS; Charles River Labs) or 2.5% charcoal-dextran stripped FBS (Atlanta Biologicals, Cat# S11650) to avoid interference from GCs in fetal bovine serum. Undiluted FBS contains approximately 0.15 μg/dL cortisol, 100× less than normal human morning serum cortisol (normal range 10–20 μg/dL) [33]. Thus the cortisol concentrations after 40-fold dilution of FBS into tissue culture media would not be expected to have any activity. FBS 2.5% was used in later experiments due to better cell growth, better reproducibility, and closer resemblance to xenograft conditions. All error bars represent the standard error across 4 biological replicates. Cells were grown in either dexamethasone (high GR specificity) or cortisol (physiologically relevant).

### *In vitro* viability assays

For viability assays, tumor cells were seeded in 96-well plates in a volume of 180 μL at a density designed to achieve 3–5 doublings during the incubation period. Dexamethasone (Sigma), cortisol (Sigma), and relacorilant (Corcept Therapeutics) were added 1 day later, and cytotoxic agents were added an additional day after that. Cells were incubated for an additional 3 days before assaying with CellTiter-Glo^®^ (Promega). High (untreated) and low (no plated cells) controls for each plate were used for normalization and to calculate residual viability using the formula: Residual viability = (Max viability of cytotoxic agent at top concentrations)/(High control – low control) × 100. Curve fits were conducted in GraphPad Prism (RRID:SCR_002798) using a 4-parameter fit. Media were removed and 200 μL of CellTiter-Glo reagent were added to each well. The plate was shaken for 2 minutes and then left to equilibrate for 10 minutes prior to reading luminescence on the Biotech Synergy II microplate reader.

### *In vitro* apoptosis assays

For kinetic image-based assays, tumor cells were seeded in a volume of 180 μL with Incucyte^®^ NucLight Rapid Red (Essen Biosciences) and Incucyte Caspase 3/7 reagent (Essen Biosciences). One to three images/day/well were acquired using an Incucyte S3 live cell imaging system (Essen Biosciences). Image acquisition began 1 day after seeding and coincided with the addition of cortisol/relacorilant (day 1; 0 h). Paclitaxel (Sigma) was added on day 2 (24 h). Image analysis was conducted using the Incucyte Analysis Software (Essen Biosciences) to determine the number of cells positive for each fluorescence marker. The apoptotic index was calculated by dividing the number of caspase 3/7 positive cells by the total number of NucLight positive cells.

### *In vivo* studies

All studies were conducted following an approved institutional animal care and use committee (IACUC) protocol. All experimental data management and reporting procedures were in strict accordance with applicable Crown Bioscience Guidelines and Standard Operating Procedures. All study procedures at Crown Bioscience China were conducted by qualified personnel and were in accordance with the approved IACUC protocol and Crown Bioscience China Standard Operating Procedures.

Blinding and power analysis were not applicable to these studies.

### Xenograft studies in mice

In all xenograft studies, mice were handled and acclimatized in a manner intended to avoid altering normal GC levels. Mice were not dosed with cortisol, corticosterone, or any other GR agonist. Tumors were measured by caliper and mice were randomized when tumor volumes were 100–200 mm^3^. There were 10 animals/group, and error bars represent the standard error within each group.

### MIA PaCa-2 xenografts

3 × 10^6^ MIA PaCa-2 (pancreatic) cells were implanted in the right flank of female Balb/c nude mice (Shanghai Laboratory Animal Center) in 100 μL phosphate-buffered saline (PBS) mixed 1:1 with Matrigel^®^ (Corning). Treatment began upon randomization with relacorilant 30 mg/kg formulated in 10% DMSO/0.1% Tween^®^ 80/89.9% HPMC (0.5%) and/or paclitaxel (Beijing Union Pharmaceutical Factory) in 0.9% sodium chloride solution. Paclitaxel was dosed once every four days (Q4D) via intravenous injection, and relacorilant was dosed the day prior and day of paclitaxel administration by oral gavage. The IACUC protocol numbers AN-1702-013-857 and AN-1507-009-615 were approved by the Crown Bioscience ethics committee.

### HeLa xenograft

5 × 10^6^ HeLa (cervical) cells were implanted in the right flank of female Balb/c nude mice (HFK Bioscience Co) in 100 μL PBS mixed 1:1 with Matrigel (Corning). Treatment began upon randomization with relacorilant 30 mg/kg formulated in 10% DMSO/0.1% Tween 80/89.9% HPMC (0.5%) and/or paclitaxel (Beijing Xiehe Pharmaceutical Factory) in 0.9% sodium chloride solution. Paclitaxel was dosed Q4D via intravenous injection, and relacorilant was dosed the day prior and day of paclitaxel administration by oral gavage. The IACUC protocol number AN-1507-009-1043 was approved by the Crown Bioscience ethics committee.

### CC6279 xenograft

Fresh tumor tissues from mice bearing established CC6279 (cholangiocarcinoma) patient-derived tumors (Crown Bioscience) were harvested and cut into small pieces (approximately 2–3 mm in diameter). Tumor fragments were implanted in the right flank of female Balb/c nude mice (Beijing Anikeeper Biotech) in 100 μL PBS mixed 1:1 with Matrigel (Corning). Treatment began upon randomization with relacorilant 30 mg/kg formulated in 10% DMSO/0.1% Tween 80/89.9% HPMC (0.5%) and/or paclitaxel (Beijing Xiehe Pharmaceutical Factory) in 0.9% sodium chloride solution. Paclitaxel was dosed Q4D via intravenous injection, and relacorilant was dosed daily by oral gavage. Upon termination, tumors were excised and fixed in 10% neutral buffered formalin then paraffin embedded. The IACUC protocol number AN- AN-1903-05-988 was approved by the Crown Bioscience ethics committee.

### Immunohistochemistry

4–5 μm sections of paraffin-embedded tumor tissue were mounted on glass slides. Primary antibodies included CK18 (EPR17347, Abcam), Ki67 (SP6, Abcam or 12202S, Cell Signaling Technologies), and cleaved-caspase-3 (9664s, Cell Signaling Technologies) and were all of rabbit origin. A rabbit-isotype control was also included to confirm specificity. Anti-rabbit Poly-HRP-IgG (Bond Polymer, Leica) secondary antibody detection kits were used. All images were counterstained with hematoxylin (Sigma, HHS128-4L). Images were captured at 40× resolution on a Pannoramic^®^ Scan II whole-slide scanner (3DHistech). H-scores were determined in fields pre-selected to contain only tumors using the HALO platform (Indica Labs) using the formula H-Score = (% at 0) × 0 + (% at 1) × 1 + (% at 2) × 2 + (% at 3) × 3 where “at 0–3” represents the intensity of staining.

### Statistics

Two-tailed, unpaired *T*-tests were performed between noted treatments using GraphPad Prism and Microsoft^®^ Excel^®^. For kinetic experiments, the terminal timepoint was used for statistical analysis. Normality tests were performed, and parametric *T*-tests were used when normality criteria were met. Otherwise, non-parametric tests were used.

## References

[1] Cannataro V , Gaffney S , Stender C , Zhan Z , Philips M , Greenstein A , Townsend J . The likelihood of heterogeneity or additional mutation to compromise targeting of KRAS G12C. J Thorac Oncol. 2017; 12:S1538. 10.1016/j.jtho.2017.06.029.

[2] Arumugam T , Ramachandran V , Fournier KF , Wang H , Marquis L , Abbruzzese JL , Gallick GE , Logsdon CD , McConkey DJ , Choi W . Epithelial to mesenchymal transition contributes to drug resistance in pancreatic cancer. Cancer Res. 2009; 69:5820–28. 10.1158/0008-5472.CAN-08-2819. 19584296 PMC4378690

[3] Sharma SV , Lee DY , Li B , Quinlan MP , Takahashi F , Maheswaran S , McDermott U , Azizian N , Zou L , Fischbach MA , Wong KK , Brandstetter K , Wittner B , et al. A chromatin-mediated reversible drug-tolerant state in cancer cell subpopulations. Cell. 2010; 141:69–80. 10.1016/j.cell.2010.02.027. 20371346 PMC2851638

[4] Melhem A , Yamada SD , Fleming GF , Delgado B , Brickley DR , Wu W , Kocherginsky M , Conzen SD . Administration of glucocorticoids to ovarian cancer patients is associated with expression of the anti-apoptotic genes SGK1 and MKP1/DUSP1 in ovarian tissues. Clin Cancer Res. 2009; 15:3196–204. 10.1158/1078-0432.CCR-08-2131. 19383827 PMC4707040

[5] Zhang C , Beckermann B , Kallifatidis G , Liu Z , Rittgen W , Edler L , Büchler P , Debatin KM , Büchler MW , Friess H , Herr I . Corticosteroids induce chemotherapy resistance in the majority of tumour cells from bone, brain, breast, cervix, melanoma and neuroblastoma. Int J Oncol. 2006; 29:1295–301. 17016664

[6] Zhang C , Kolb A , Büchler P , Cato AC , Mattern J , Rittgen W , Edler L , Debatin KM , Büchler MW , Friess H , Herr I . Corticosteroid co-treatment induces resistance to chemotherapy in surgical resections, xenografts and established cell lines of pancreatic cancer. BMC Cancer. 2006; 6:61. 10.1186/1471-2407-6-61. 16539710 PMC1434760

[7] Wang JC , Harris CL . Glucocorticoid signaling. In: Wang JC and Harris C , eds. Adv Exp Med Biol. (New York, NY: Springer New York). 2015; 2895–98.

[8] Nicolaides NC , Kino T , Roberts ML , Katsantoni E , Sertedaki A , Moutsatsou P , Psarra AG , Chrousos GP , Charmandari E . The Role of S-Palmitoylation of the Human Glucocorticoid Receptor (hGR) in Mediating the Nongenomic Glucocorticoid Actions. J Mol Biochem. 2017; 6:3–12. 28775968 PMC5538142

[9] Miller AH , Spencer RL , Pearce BD , Pisell TL , Azrieli Y , Tanapat P , Moday H , Rhee R , McEwen BS . Glucocorticoid receptors are differentially expressed in the cells and tissues of the immune system. Cell Immunol. 1998; 186:45–54. 10.1006/cimm.1998.1293. 9637764

[10] Rogatsky I , Hittelman AB , Pearce D , Garabedian MJ . Distinct glucocorticoid receptor transcriptional regulatory surfaces mediate the cytotoxic and cytostatic effects of glucocorticoids. Mol Cell Biol. 1999; 19:5036–49. 10.1128/MCB.19.7.5036. 10373553 PMC84339

[11] Gruver-Yates AL , Cidlowski JA . Tissue-specific actions of glucocorticoids on apoptosis: a double-edged sword. Cells. 2013; 2:202–23. 10.3390/cells2020202. 24709697 PMC3972684

[12] Warris LT , van den Heuvel-Eibrink MM , Ariës IM , Pieters R , van den Akker EL , den Boer ML . Hydrocortisone does not influence glucocorticoid sensitivity of acute lymphoblastic leukemia cells. Haematologica. 2015; 100:e137–9. 10.3324/haematol.2014.112177. 25425687 PMC4380735

[13] Longui CA , Santos MC , Formiga CB , Oliveira DV , Rocha MN , Faria CD , Kochi C , Monte O . Antiproliferative and apoptotic potencies of glucocorticoids: nonconcordance with their antiinflammatory and immunosuppressive properties. Arq Bras Endocrinol Metabol. 2005; 49:378–83. 10.1590/s0004-27302005000300008. 16543991

[14] Skor MN , Wonder EL , Kocherginsky M , Goyal A , Hall BA , Cai Y , Conzen SD . Glucocorticoid receptor antagonism as a novel therapy for triple-negative breast cancer. Clin Cancer Res. 2013; 19:6163–72. 10.1158/1078-0432.CCR-12-3826. 24016618 PMC3860283

[15] Stringer-Reasor EM , Baker GM , Skor MN , Kocherginsky M , Lengyel E , Fleming GF , Conzen SD . Glucocorticoid receptor activation inhibits chemotherapy-induced cell death in high-grade serous ovarian carcinoma. Gynecol Oncol. 2015; 138:656–62. 10.1016/j.ygyno.2015.06.033. 26115975 PMC4556542

[16] Moran TJ , Gray S , Mikosz CA , Conzen SD . The glucocorticoid receptor mediates a survival signal in human mammary epithelial cells. Cancer Res. 2000; 60:867–72. 10706096

[17] Hunt HJ , Belanoff JK , Walters I , Gourdet B , Thomas J , Barton N , Unitt J , Phillips T , Swift D , Eaton E . Identification of the Clinical Candidate (R)-(1-(4-Fluorophenyl)-6-((1-methyl-1H-pyrazol-4-yl)sulfonyl)-4,4a,5,6,7,8-hexahydro-1H-pyrazolo[3,4-g]isoquinolin-4a-yl)(4-(trifluoromethyl)pyridin-2-yl)methanone (CORT125134): A Selective Glucocorticoid Receptor (GR) Antagonist. J Med Chem. 2017; 60:3405–21. 10.1021/acs.jmedchem.7b00162. 28368581

[18] Hunt H , Donaldson K , Strem M , Zann V , Leung P , Sweet S , Connor A , Combs D , Belanoff J . Assessment of Safety, Tolerability, Pharmacokinetics, and Pharmacological Effect of Orally Administered CORT125134: An Adaptive, Double-Blind, Randomized, Placebo-Controlled Phase 1 Clinical Study. Clin Pharmacol Drug Dev. 2018; 7:408–21. 10.1002/cpdd.389. 28967708 PMC5947602

[19] Munster PN , Sachdev JC , Fleming GF , Borazanci EH , Grabowsky JA , Sharma M . Relacorilant (RELA) with nab-paclitaxel (NP): safety and activity in patients with pancreatic ductal adenocarcinoma (PDAC) and ovarian cancer (OvCA). J Clin Oncol. 2019; 37:4130. 10.1200/JCO.2019.37.15_suppl.4130.

[20] Terzolo M , Iacuaniello D , Pia A , Adriano P , Moraitis A , Pivonello R . SUN-463 tumor shrinkage with preoperative relacorilant therapy in two patients with Cushing disease due to pituitary macroadenomas. J Endocrine Soc. 2019; 3:SUN-463. https://doi.org/10.1210%2Fjs.2019-SUN-463.

[21] Mayo Clinic Laboratories. Cortisol, serum Available at: https://endocrinology.testcatalog.org/show/CORT. Accessed 3 17, 2021.

[22] Pang D , Kocherginsky M , Krausz T , Kim SY , Conzen SD . Dexamethasone decreases xenograft response to Paclitaxel through inhibition of tumor cell apoptosis. Cancer Biol Ther. 2006; 5:933–40. 10.4161/cbt.5.8.2875. 16775428

[23] West DC , Kocherginsky M , Tonsing-Carter EY , Dolcen DN , Hosfield DJ , Lastra RR , Sinnwell JP , Thompson KJ , Bowie KR , Harkless RV , Skor MN , Pierce CF , Styke SC , et al. Discovery of a Glucocorticoid Receptor (GR) Activity Signature Using Selective GR Antagonism in ER-Negative Breast Cancer. Clin Cancer Res. 2018; 24:3433–46. 10.1158/1078-0432.CCR-17-2793. 29636357 PMC6530562

[24] Kroon J , Puhr M , Buijs JT , van der Horst G , Hemmer DM , Marijt KA , Hwang MS , Masood M , Grimm S , Storm G , Metselaar JM , Meijer OC , Culig Z , van der Pluijm G . Glucocorticoid receptor antagonism reverts docetaxel resistance in human prostate cancer. Endocr Relat Cancer. 2016; 23:35–45. 10.1530/ERC-15-0343. 26483423 PMC4657186

[25] Yamamoto K , Ichijo H , Korsmeyer SJ . BCL-2 is phosphorylated and inactivated by an ASK1/Jun N-terminal protein kinase pathway normally activated at G(2)/M. Mol Cell Biol. 1999; 19:8469–78. 10.1128/MCB.19.12.8469. 10567572 PMC84954

[26] D'Antona L , Dattilo V , Catalogna G , Scumaci D , Fiumara CV , Musumeci F , Perrotti G , Schenone S , Tallerico R , Spoleti CB , Costa N , Iuliano R , Cuda G , et al. In Preclinical Model of Ovarian Cancer, the SGK1 Inhibitor SI113 Counteracts the Development of Paclitaxel Resistance and Restores Drug Sensitivity. Transl Oncol. 2019; 12:1045–55. 10.1016/j.tranon.2019.05.008. 31163384 PMC6545392

[27] Liu L , Aleksandrowicz E , Schönsiegel F , Gröner D , Bauer N , Nwaeburu CC , Zhao Z , Gladkich J , Hoppe-Tichy T , Yefenof E , Hackert T , Strobel O , Herr I . Dexamethasone mediates pancreatic cancer progression by glucocorticoid receptor, TGFβ and JNK/AP-1. Cell Death Dis. 2017; 8:e3064. 10.1038/cddis.2017.455. 28981109 PMC5680577

[28] Li Z , Chen Y , Cao D , Wang Y , Chen G , Zhang S , Lu J . Glucocorticoid up-regulates transforming growth factor-beta (TGF-beta) type II receptor and enhances TGF-beta signaling in human prostate cancer PC-3 cells. Endocrinology. 2006; 147:5259–67. 10.1210/en.2006-0540. 16887915

[29] Abukiwan A , Nwaeburu CC , Bauer N , Zhao Z , Liu L , Gladkich J , Gross W , Benner A , Strobel O , Fellenberg J , Herr I . Dexamethasone-induced inhibition of miR-132 via methylation promotes TGF-β-driven progression of pancreatic cancer. Int J Oncol. 2019; 54:53–64. 10.3892/ijo.2018.4616. 30387838 PMC6255064

[30] Hangauer MJ , Viswanathan VS , Ryan MJ , Bole D , Eaton JK , Matov A , Galeas J , Dhruv HD , Berens ME , Schreiber SL , McCormick F , McManus MT . Drug-tolerant persister cancer cells are vulnerable to GPX4 inhibition. Nature. 2017; 551:247–50. 10.1038/nature24297. 29088702 PMC5933935

[31] Keith BD . Systematic review of the clinical effect of glucocorticoids on nonhematologic malignancy. BMC Cancer. 2008; 8:84. 10.1186/1471-2407-8-84. 18373855 PMC2330150

[32] Barretina J , Caponigro G , Stransky N , Venkatesan K , Margolin AA , Kim S , Wilson CJ , Lehár J , Kryukov GV , Sonkin D , Reddy A , Liu M , Murray L , et al. The Cancer Cell Line Encyclopedia enables predictive modelling of anticancer drug sensitivity. Nature. 2012; 483:603–07. 10.1038/nature11003. 22460905 PMC3320027

[33] Cao Z , West C , Norton-Wenzel CS , Rej R , Davis FB , Davis PJ , Rej R . Effects of resin or charcoal treatment on fetal bovine serum and bovine calf serum. Endocr Res. 2009; 34:101–08. 10.3109/07435800903204082. 19878070

